# Assessing Knowledge and Awareness of Epilepsy Surgery in Healthcare Students and Interns at King Abdulaziz University

**DOI:** 10.1155/bn/4417001

**Published:** 2026-10-04

**Authors:** Soha A. Alomar, Abdullah M. Wali, Duaa M. Alshabani, Thamer H. Alsharif, Abdulrahman J. Sabbagh

**Affiliations:** ^1^ Faculty of Medicine Department of Surgery Division of Neurosurgery King Abdulaziz University Jeddah Saudi Arabia kau.edu.sa; ^2^ King Abdulaziz University Jeddah Saudi Arabia kau.edu.sa; ^3^ Departmet of Surgery Division of Neurosurgery King Abdulaziz Specialist Hospital Taif Saudi Arabia

**Keywords:** drug-resistant epilepsy, epilepsy surgery, healthcare students, knowledge and awareness, medical education

## Abstract

**Objectives:**

The objective of this study is to assess the knowledge and awareness of epilepsy surgery among healthcare students and interns at King Abdulaziz University. By identifying gaps in understanding and perception, the study is aimed at informing future educational strategies to enhance awareness of surgical options for epilepsy management.

**Methods:**

Data were collected using an online self‐administered questionnaire assessing participants′ awareness and knowledge of epilepsy surgery, drug‐resistant epilepsy (DRE), surgical referral timing, and perceptions regarding the risks and benefits of epilepsy surgery.

**Results:**

Among 500 participants, only 25.8% (including 50.7% of medical students) knew that epilepsy could be treated surgically. Just 10.4% (17.3% of medical students) felt their medical training adequately covered epilepsy surgery. Only 10.6% (24.7% of medical students) correctly identified partial/focal epilepsy as surgically treatable, and 14.6% (30% of medical students) believed the benefits of surgery were high. “DRE” was correctly recognized by 44.2% overall (30% of medical students). Medical students had the highest knowledge scores (12.33 ± 4.04), followed by nursing (10.71 ± 2.87), pharmacy (9.94 ± 2.12), and laboratory students (9.85 ± 1.52).

**Conclusion:**

A significant knowledge gap exists regarding epilepsy surgery among healthcare students and interns. Medical students demonstrated greater awareness than their nonmedical peers. Enhancing medical and allied health curricula could improve future referral practices and patient outcomes.

## 1. Introduction

Epilepsy is a chronic neurological disease characterized by recurrent unprovoked seizures resulting from abnormal excessive neuronal activity in the brain. It has various causes and is one of the most prevalent neurological conditions in Saudi Arabia, with a reported prevalence of 6.54 per 1000 individuals [1, 2].

Despite advances in antiseizure medications (ASMs), more than 30% of epilepsy patients have progressed to drug‐resistant epilepsy (DRE), defined as the “failure of adequate trials of two tolerated and appropriately chosen and used ASMs regimens to achieve seizure freedom” [3–5].

Epilepsy surgery is a therapeutic option for patients with DRE [6, 7]. It not only improves seizure control but also enhances quality of life, reduces healthcare costs, and lowers the risk of premature death [8].

Two major randomized controlled trials have demonstrated the superiority of combining surgery with ASMs over ASM therapy alone for treating refractory mesial temporal lobe epilepsy [6, 7]. However, despite these benefits, surgical treatment for epilepsy remains underutilized and is often delayed [8, 9]. A major contributor to this delay is the lack of adherence to referral guidelines, often due to insufficient awareness among healthcare professionals [10]. The International League Against Epilepsy (ILAE) recommends that all patients with DRE should be referred to a comprehensive epilepsy center for surgical candidacy evaluation once adequate trials of two appropriately selected and tolerated ASMs have failed to achieve seizure freedom [4]. Despite these recommendations, referral for epilepsy surgery evaluation remains substantially delayed worldwide.

A 2013 study found that 56% of Saudi healthcare professionals were unaware that surgery could be a treatment option for epilepsy [11]. Interdisciplinary collaboration is essential in the management of epilepsy patients. Healthcare students and interns—across fields such as medicine, nursing, pharmacy, and laboratory sciences—can play a vital role in this collaborative care model. Although epilepsy surgery is a highly specialized field, baseline awareness regarding surgically treatable epilepsy and referral pathways remains important for healthcare trainees. By participating in epilepsy care during training, they can gain practical insights into surgical treatment, preparing them for future professional roles. Despite increasing evidence supporting epilepsy surgery for DRE, little is known about the level of awareness among multidisciplinary healthcare students and interns in Saudi Arabia. Previous studies have largely focused on neurologists or medical students, leaving important gaps regarding allied healthcare disciplines and the influence of clinical neurology and neurosurgery training on knowledge of epilepsy surgery and referral practices. Therefore, this study addresses an important educational and clinical gap by evaluating knowledge and awareness of epilepsy surgery among healthcare students and interns from multiple disciplines at King Abdulaziz University. The findings may help guide curriculum development and interdisciplinary educational strategies aimed at improving early recognition and referral of patients with DRE.

This study is aimed at providing insight into the levels of knowledge and awareness of epilepsy surgery among healthcare students and interns at King Abdulaziz University in Jeddah, Saudi Arabia, and analyzing the factors influencing their understanding of this treatment modality. It provides a multidisciplinary assessment of epilepsy surgery awareness among healthcare students and interns and explores factors associated with knowledge, including specialty and clinical neurology/neurosurgery exposure.

## 2. Materials and Methods

### 2.1. Study Design

This cross‐sectional descriptive study was conducted among healthcare students and interns at King Abdulaziz University, Jeddah, Saudi Arabia. The study population included undergraduate students from the colleges of medicine, nursing, pharmacy, and medical laboratory sciences, which consisted of students enrolled in the medical laboratory sciences program, in addition to medical interns. In the Saudi medical education system, “interns” are medical graduates completing a mandatory supervised clinical training year prior to independent medical practice or residency training. These disciplines were selected because of their potential involvement in multidisciplinary epilepsy care and patient referral pathways, although their levels of direct clinical exposure vary. Nonhealthcare and nonclinical university programs were not included in the study. Ethical approval was obtained from the Research Ethics Committee of King Abdulaziz University Hospital, Jeddah, Saudi Arabia (NCBE Registration No. HA‐02‐J‐008), on January 11, 2024. A self‐administered questionnaire was used, and participation was voluntary. Respondents were required to complete all questions. A convenience sampling method was used, and the questionnaire was distributed electronically among eligible healthcare students and interns. This sampling approach was adopted to recruit all eligible healthcare students and interns who were willing to participate during the study period. An a priori sample size calculation was performed. For descriptive cross‐sectional studies, the commonly used sample size formula is *n* = *Z* ^2^ *P*(1 − *P*)/*d* ^2^, where *n* is the required sample size, *Z* is the standard normal deviate, *P* is the estimated prevalence, and *d* is the acceptable margin of error. The required sample size was 385 participants. A total of 500 students and interns from various healthcare disciplines and stages of education participated, providing a diverse sample for assessing knowledge and attitudes toward epilepsy surgery. Inclusion criteria included healthcare students and interns enrolled at King Abdulaziz University during the study period who agreed to participate voluntarily. Participants from medicine, nursing, pharmacy, and laboratory sciences were included. Incomplete questionnaires were excluded from analysis.

### 2.2. Preparation of Questionnaire

The questionnaire was developed by AW, DA, and TH after reviewing previously published studies assessing awareness and knowledge of epilepsy surgery among healthcare professionals and students [12–14]. The survey collected demographic and academic information, including educational level and healthcare specialty, and assessed participants′ understanding of epilepsy management and epilepsy surgery. Questionnaire items addressing DRE, referral timing, and surgical candidacy were developed in accordance with the ILAE definitions and recommendations regarding DRE criteria and epilepsy surgery evaluation. The questionnaire was reviewed by the principal investigator, consultant neurosurgeons, and medical education researchers to assess content validity, face validity, clarity, relevance, and comprehensiveness, and minor modifications were made to improve readability and suitability for the target population. The questionnaire was administered in English, the official language of medical education at the institution. Because the instrument was adapted from previously published questionnaires rather than developed de novo, formal psychometric validation, including construct validity, internal consistency testing (Cronbach′s alpha), and test–retest reliability, was not performed.

Knowledge scores ranged from 0 to 36 and were categorized as low (0–12), moderate (13–24), and high (25–36). Scoring and score categorization were adapted from a previously published Saudi study assessing awareness of epilepsy surgery among medical students at Umm Al‐Qura University [14]. Correct answers were assigned 2 points, “unknown” responses 1 point, and incorrect answers 0 points. Questions with multiple correct answers were awarded 2 points for each correct response. Although Bloom′s categorization was considered, the original scoring classification was retained to maintain methodological consistency and comparability with regional data.

### 2.3. Data Collection

The survey consisted of three sections. The first obtained informed consent. The second collected demographic and academic information. The third included 19 questions measuring knowledge and awareness of epilepsy surgery. The questionnaire was administered electronically and distributed to eligible students and interns between January and April 2024 using online platforms. The survey link was shared through institutional student communication channels, electronic messaging platforms, and student social media groups to facilitate participation across the included healthcare disciplines. Participation was voluntary, and responses were collected anonymously.

### 2.4. Statistical Analysis

Data were analyzed using SPSS Version 22 (IBM Corp., Armonk, New York, United States). Categorical variables were presented as frequencies and percentages. The distribution of knowledge scores was assessed using the Shapiro–Wilk test, which demonstrated a mild deviation from normality. Therefore, nonparametric tests were used for group comparisons. Continuous variables were summarized as mean ± standard deviation (SD) for descriptive purposes because of the large sample size, whereas the Mann–Whitney *U* test was used to compare two groups, and the Kruskal–Wallis test was used for comparisons among more than two groups. Pearson′s chi‐square test was used to analyze categorical variables. A *p* value < 0.05 was considered statistically significant.

## 3. Results

### 3.1. Results

A total of 500 students participated in this study. Table 1 presents their demographic characteristics. The age of the participants ranged from 18 to 33 years, with a mean age of 22.83 years. Females slightly outnumbered males (54.4% vs. 45.6%). The sample consisted of students from various colleges, including medical students (*n* = 150, 30.0%), nursing students (*n* = 151, 30.2%), pharmacy students (*n* = 119, 23.8%), and laboratory students (*n* = 80, 16.0%). The distribution of students across college years was as follows: 1st year (*n* = 19, 3.8%), 2nd year (*n* = 46, 9.2%), 3rd year (*n* = 70, 14.0%), 4th year (*n* = 79, 15.8%), 5th year (*n* = 53, 10.6%), 6th year (*n* = 51, 10.2%), and interns (*n* = 182, 36.2%). Most participants were in clinical years compared with preclinical years (83.6% vs. 16.4%). Among all participants, 110 (22.0%) had completed a neurology or neurosurgery rotation, whereas 274 (54.8%) had not yet completed the rotation. The question regarding completion of neurology or neurosurgery rotations was not applicable to 116 (23.2%) participants who were in the preclinical years.

**Table 1:** Demographic characteristics of participants (*n* = 500).

Characteristics	Value
Age (years)	22.83 ± 1.88 (18.00–33.00)
Sex	
Male	228 (45.6%)
Female	272 (54.4%)
Specialty	
Medical students	150 (30.0%)
Nursing students	151 (30.2%)
Pharmacy students	119 (23.8%)
Laboratory students	80 (16.0%)
College year	
1st year	19 (3.8%)
2nd year	46 (9.2%)
3rd year	70 (14.0%)
4th year	79 (15.8%)
5th year	53 (10.6%)
6th year	51 (10.2%)
Intern	182 (36.4%)
Academic year	
Preclinical years	82 (16.4%)
Clinical years	418 (83.6%)
Completed a neurology or neurosurgery rotation	
Yes	110 (22.0%)
No	274 (54.8%)
Not applicable	116 (23.2%)

### 3.2. Awareness

Participants′ responses to the awareness section across different colleges are shown in Table 2. Most participants (86.6%) did not have or know someone with epilepsy. Only 25.8% were aware that epilepsy can be treated surgically. A majority (66.6%) reported not receiving any information about epilepsy surgery; 11.0% received information from the medical school curriculum, 10.0% read about it, 6.2% gained knowledge through general sources, 4.2% had seen a case treated surgically, and 1.0% each learned from a neurologist or family members who had undergone surgery. Regarding education on epilepsy surgery, 10.4% believed it was sufficient, 27.6% did not, and 62.0% were unsure.

**Table 2:** Awareness about epileptic surgery among different colleges.

Statements	All participants (*n* = 500)	Medical (*n* = 150)	Nursing (*n* = 151)	Pharmacy (*n* = 119)	Laboratory (*n* = 80)
Do you have/know someone with epilepsy?					
No	433 (86.6%)	111 (74.0%)	132 (87.4%)	115 (96.6%)	75 (93.8%)
Yes	67 (13.4%)	39 (26.0%)	19 (12.6%)	4 (3.4%)	5 (6.2%)
Have you heard that epilepsy can be treated surgically?					
No	371 (74.2%)	74 (49.3%)	115 (76.2%)	106 (89.1%)	76 (95.0%)
Yes	129 (25.8%)	76 (50.7%)	36 (23.8%)	13 (10.9%)	4 (5.0%)
Where did you get the information about epilepsy surgery?					
Never heard about it	333 (66.6%)	61 (40.7%)	96 (63.6%)	101 (84.9%)	75 (93.8%)
In medical school curriculum	55 (11.0%)	43 (28.7%)	6 (4.0%)	6 (5.0%)	—
I read about it	50 (10.0%)	16 (10.7%)	24 (15.9%)	8 (6.7%)	2 (2.5%)
General knowledge	31 (6.2%)				
I have seen a case treated surgically	21 (4.2%)	10 (6.7%)	10 (6.6%)	1 (0.8%)	—
From my neurologist	5 (1.0%)	1 (0.7%)	3 (2.0%)	—	1 (1.2%)
Family members who had epilepsy surgery	5 (1.0%)	3 (2.0%)	2 (1.3%)	—	—
Do you believe there is sufficient education on epileptic surgery during your medical training?					
Yes	52 (10.4%)	26 (17.3%)	21 (13.9%)	2 (1.7%)	3 (3.8%)
No	138 (27.6%)	60 (40.0%)	38 (25.2%)	26 (21.8%)	14 (17.5%)
Unsure	310 (62.0%)	64 (42.7%)	92 (60.9%)	91 (76.5%)	63 (78.8%)

### 3.3. Knowledge

Participants′ responses to the knowledge section across colleges are shown in Table 3. Only 18.2% correctly identified that surgical treatment should be considered in selected epilepsy cases. Just 10.6% knew partial or focal epilepsy could be treated surgically, whereas 79.6% did not know. Only 2.4% correctly identified the risk of epilepsy surgery as moderate, with 69.0% unsure. Regarding potential benefits, 18% perceived it as high and very high, whereas 70.2% were unsure.

**Table 3:** Knowledge about epileptic surgery among different colleges.

Statements	All participants (*n* = 500)	Medical (*n* = 150)	Nursing (*n* = 151)	Pharmacy (*n* = 119)	Laboratory (*n* = 80)
When do you think surgical treatment for epilepsy should be considered?					
Always	24 (4.8%)	8 (5.3%)	14 (9.3%)	1 (0.8%)	1 (1.2%)
Never	4 (0.8%)	—	3 (2.0%)	1 (0.8%)	—
In selected cases (correct answer)	91 (18.2%)	55 (36.7%)	26 (17.2%)	8 (6.7%)	2 (2.5%)
Only as a last resort	53 (10.6%)	36 (24.0%)	8 (5.3%)	6 (5.0%)	3 (3.8%)
I do not know	328 (65.6%)	51 (34.0%)	100 (66.2%)	103 (86.6%)	74 (92.5%)
What type of epilepsy can be surgically treated?					
Generalized epilepsy (correct answer)	21 (4.2%)	9 (5.0%)	10 (6.6%)	1 (0.8%)	1 (1.2%)
Partial or focal epilepsy (correct answer)	53 (10.6%)	37 (24.7%)	11 (7.3%)	3 (2.5%)	2 (2.5%)
Neither	10 (2.0%)	3 (2.0%)	6 (4.0%)	1 (0.8%)	—
Both	18 (3.6%)	11 (7.3%)	4 (2.6%)	2 (1.7%)	1 (1.2%)
I do not know	398 (79.6%)	90 (60.0%)	120 (79.5%)	112 (94.1%)	76 (95.0%)
What is your impression of the risk of epilepsy surgery?					
Low (correct answer)	12 (2.4%)	5 (3.3%)	5 (3.3%)	1 (0.8%)	1 (1.2%)
Moderate	37 (7.4%)	20 (13.3%)	14 (9.3%)	1 (0.8%)	2 (2.5%)
High	69 (13.8%)	35 (23.3%)	18 (11.9%)	13 (10.9%)	3 (3.8%)
Very high	37 (7.4%)	19 (12.7%)	14 (9.3%)	3 (2.5%)	1 (1.2%)
I do not know	345 (69.0%)	71 (47.3%)	100 (66.2%)	101 (84.9%)	73 (91.2%)
What is your impression of the potential benefit of epilepsy surgery?					
Low	10 (2.0%)	3 (2.0%)	6 (4.0%)	—	1 (1.2%)
Moderate	49 (9.8%)	21 (14.0%)	19 (12.6%)	6 (5.0%)	3 (3.8%)
High (correct answer)	73 (14.6%)	45 (30.0%)	19 (12.6%)	6 (5.0%)	3 (3.8%)
Very high (correct answer)	17 (3.4%)	8 (5.3%)	6 (4.0%)	3 (2.5%)	—
I do not know	351 (70.2%)	73 (48.7%)	101 (66.9%)	104 (87.4%)	73 (91.2%)
Are you familiar with the term of “drug‐resistant epilepsy DRE”?					
No	354 (70.8%)	66 (44.0%)	112 (74.2%)	101 (84.9%)	75 (93.8%)
Yes (correct answer)	146 (29.2%)	84 (56.0%)	39 (25.8%)	18 (15.1%)	5 (6.2%)
Have you ever heard of mesial temporal/hippocampal sclerosis?					
No	447 (89.4%)	122 (81.3%)	132 (87.4%)	115 (96.6%)	78 (97.5%)
Yes	53 (10.6%)	28 (18.7%)	19 (12.6%)	4 (2.4%)	2 (2.5%)
What constitutes drug resistant epilepsy?					
Failure of seizure control after one ASMs (monotherapy or polytherapy)	104 (20.8%)	23 (15.3%)	34 (22.5%)	23 (19.3%)	24 (30.0%)
Failure of seizure control after two ASMs (monotherapy or polytherapy) (correct answer)	221 (44.2%)	45 (30.0%)	75 (49.7%)	63 (52.9%)	38 (47.5%)
Failure of seizure control after three and more ASMs (monotherapy or polytherapy)	175 (35.0%)	82 (54.7%)	42 (27.8%)	33 (27.7%)	18 (22.5%)
Patient who has drug resistant epilepsy should be referred to?					
To neurosurgeon and neurologist (correct answer)	163 (32.6%)	96 (64.0%)	44 (29.1%)	13 (10.9%)	10 (12.5%)
To radiologist	22 (4.4%)	8 (5.3%)	12 (7.9%)	3 (1.7%)	—
I do not know	315 (63.0%)	46 (30.7%)	95 (62.9%)	104 (87.4%)	70 (87.5%)
Epileptic surgery is typically considered when					
First‐line medications fail (correct answer)	124 (24.8%)	37 (24.7%)	33 (21.9%)	25 (21.0%)	29 (36.2%)
A patient experiences their first seizure	132 (26.4%)	11 (7.3%)	54 (35.8%)	46 (38.7%)	21 (26.2%)
As an alternative to any antiseizure medications	143 (28.6%)	82 (54.7%)	30 (19.9%)	19 (16.0%)	12 (15.0%)
In cases of mild epilepsy only	101 (20.2%)	20 (13.3%)	34 (22.5%)	29 (24.4%)	19 (22.5%)
Which of the following techniques are you familiar with in terms of surgical treatment of epilepsy? (more than one option is accepted)					
I do not know	393 (78.6%)	91 (60.7%)	113 (74.8%)	111 (93.3%)	78 (97.5%)
Lobectomy (correct answer)	59 (11.8%)	37 (24.7%)	19 (12.6%)	3 (2.5%)	—
Hemispherectomy (correct answer)	49 (9.8%)	34 (22.7%)	13 (8.6%)	2 (1.7%)	—
Lesionectomy (correct answer)	30 (6.0%)	17 (11.3%)	9 (6.0%)	3 (2.5%)	1 (1.2%)
Corpus callosotomy (correct answer)	29 (5.8%)	15 (10.0%)	10 (6.6%)	3 (2.5%)	1 (1.2%)
Vagus nerve stimulation (correct answer)	26 (5.2%)	16 (10.7%)	7 (4.6%)	3 (2.5%)	—
Laser ablation (correct answer)	25 (5.0%)	20 (13.3%)	4 (2.6%)	1 (0.8%)	—
Neuropace (correct answer)	13 (2.6%)	6 (4.0%)	6 (4.0%)	1 (0.8%)	—
Have you ever heard sudden unexpected death in epilepsy (SUDEP)?					
Yes (correct answer)	52 (10.4%)	23 (15.3%)	21 (13.9%)	6 (5.0%)	2 (2.5%)
No	133 (26.6%)	81 (54.0%)	33 (21.9%)	11 (9.2%)	8 (10.0%)
I do not know	315 (63.0%)	46 (30.7%)	97 (64.2%)	102 (85.7%)	70 (87.5%)
Total score of knowledge (0–36)	10.88 ± 3.13 (5–26)	12.33 ± 4.04 (6–26)	10.71 ± 2.87 (5–22)	9.94 ± 2.12 (6–22)	9.85 ± 1.52 (7–17)
Level of knowledge					
Low (0–12)	408 (81.6%)	92 (61.3%)	128 (84.8%)	111 (93.3%)	77 (96.2%)
Moderate (13–24)	89 (17.8%)	55 (36.7%)	23 (15.2%)	8 (6.7%)	3 (3.8%)
High (25–36)	3 (0.6%)	3 (2.0%)	—	—	—

In terms of familiarity with specific terms, 29.2% of participants were familiar with the term “DRE,” and 10.6% had heard of mesial temporal/hippocampal sclerosis. A total of 221 participants (44.2%) correctly identified DRE as the failure of seizure control after two ASMs, whether as monotherapy or polytherapy. For the timing of referral for surgery evaluation, 14.0% said referral should be early, whereas 73.0% did not know.

Around 32.6% correctly answered that DRE patients should be referred to both neurosurgeons and neurologists, though 63.0% were unsure. Only 24.8% knew surgery is typically considered after first‐line medications fail. Familiarity with surgery techniques was low: lobectomy (11.8%), hemispherectomy (9.8%), lesionectomy (6.0%), corpus callosotomy (5.8%), vagus nerve stimulation (5.2%), laser ablation (5.0%), neuropace (2.6%), and 78.6% did not know any.

Only 10.4% of participants had heard of sudden unexpected death in epilepsy (SUDEP), whereas 63.0% had not. The total knowledge score ranged from 5 to 26, with a mean of 10.88. The total knowledge score was categorized as low (0–12) among 408 participants (81.6%), moderate (13–24) among 89 participants (17.8%), and high (25–36) among only 3 participants (0.6%).

### 3.4. Association Between the Level of Knowledge and Demographic Characteristics

Significant associations were found between the level of knowledge and participants′ specialty (*p* < 0.0001) as well as the completion of a neurology or neurosurgery rotation (*p* < 0.0001). In contrast, no significant associations were observed between the level of knowledge and sex (*p* = 0.099) or academic year (*p* = 0.396) (Table 4). Neurology and neurosurgery rotations were primarily available to medical students and, to a lesser extent, nursing students during clinical training. These rotations were generally not part of the standard curriculum for pharmacy or medical laboratory sciences students.

**Table 4:** Association between the level of knowledge and demographic characteristics.

Variables	Level of knowledge			Significance
	Low (0–12) (*n* = 408)	Moderate (13–24) (*n* = 89)	High (25–36) (*n* = 3)	
Sex				*p* = 0.099
Male (*n* = 228)	189 (46.3%)	36 (40.4%)	3 (100%)	
Female (*n* = 272)	219 (53.7%)	53 (59.6%)	—	
Specialty				*p* < 0.0001
Medical students (*n* = 150)	92 (22.5%)	55 (61.8%)	3 (100%)	
Nonmedical students (*n* = 350)	316 (77.5%)	34 (38.2%)	—	
Nursing	128 (31.4%)	23 (25.8%)	—	
Pharmacy	111 (27.2%)	8 (9.0%)	—	
Laboratory	77 (18.9%)	3 (3.4%)	—	
Academic year				*p* = 0.396
Preclinical years (*n* = 82)	63 (15.4%)	18 (20.2%)	1 (33.3%)	
Clinical years (*n* = 418)	345 (84.6%)	71 (79.8%)	2 (66.7%)	
Completed a neurology or neurosurgery rotation				*p* < 0.0001
Yes (*n* = 110)	59 (14.5%)	48 (53.9%)	3 (100.0%)	
No (*n* = 274)	243 (59.6%)	31 (34.8%)	—	
Not applicable (*n* = 116)	106 (26.0%)	10 (11.2%)	—	

### 3.5. Total Knowledge Score According to Demographic Characteristics

The total knowledge score was significantly higher among medical students compared with nonmedical students (*p* < 0.0001) and among students who had completed a neurology or neurosurgery rotation compared with those who had not completed or for whom the rotation was not applicable (*p* < 0.0001). In contrast, the total knowledge score showed no significant differences in relation to sex (*p* = 0.753) or academic year (*p* = 0.335) (Table 5). Medical students demonstrated significantly higher knowledge scores compared with nonmedical students (12.33 ± 4.04 vs. 10.25 ± 2.39, *p* < 0.0001). Similarly, participants who had completed a neurology or neurosurgery rotation showed higher knowledge scores compared with those who had not completed a rotation or for whom the rotation was not applicable (13.06 ± 3.97 vs. 10.52 ± 2.66 and 9.65 ± 2.07, respectively; *p* < 0.0001).

**Table 5:** Total knowledge score according to demographic characteristics.

Variables	Total knowledge score	Significance
Sex		*p* = 0.753
Male (*n* = 228)	10.96 ± 3.28 (5–22)	
Female (*n* = 272)	10.81 ± 3.00 (5–24)	
Specialty		*p* < 0.0001
Medical students (*n* = 150)	12.33 ± 4.04 (6–26)	
Nonmedical students (*n* = 350)	10.25 ± 2.39 (5–22)	
Nursing	10.71 ± 2.87 (5–22)	
Pharmacy	9.94 ± 2.12 (6–22)	
Laboratory	9.85 ± 1.52 (7–17)	
Academic year		*p* = 0.335
Preclinical years (*n* = 82)	11.07 ± 3.872.39 (6–26)	
Clinical years (*n* = 418)	10.84 ± 2.97 (5–26)	
Completed a neurology or neurosurgery rotation		*p* < 0.0001
Yes (*n* = 110)	13.06 ± 3.97 (7–26)	
No (*n* = 274)	10.52 ± 2.66 (5–24)	
Not applicable (*n* = 116)	9.65 ± 2.07 (8–22)	

## 4. Discussion

This study provides valuable insights into the current state of knowledge and awareness toward epilepsy surgery among healthcare students at King Abdulaziz University. By identifying gaps and areas for improvement, targeted educational interventions can be developed to enhance understanding and utilization of epilepsy surgery, ultimately improving patient care and outcomes.

Students with a deeper comprehension of epilepsy surgery are more likely to recognize DRE early and refer patients for surgical evaluation promptly, which can lead to improved outcomes and quality of life. This proactive approach is particularly crucial for primary healthcare physicians working in centers without access to neurologists, as it empowers them to make informed decisions that can significantly impact patient care. The survey revealed varying levels of awareness among the different groups, with medical students achieving the highest scores and nursing students ranking second. Differences in knowledge levels across healthcare disciplines may reflect variations in clinical exposure, curricular emphasis, and the expected degree of involvement in epilepsy surgery referral pathways and multidisciplinary epilepsy care during undergraduate training. Nevertheless, laboratory students were included in this study because multidisciplinary epilepsy care extends beyond direct physician referral pathways and involves multiple healthcare disciplines that contribute to diagnostic evaluation, perioperative care, patient counseling, and long‐term management. Although laboratory professionals are less likely to participate directly in surgical referral decisions, assessing their baseline awareness remains relevant in understanding the broader educational landscape of epilepsy care among healthcare trainees. The lower knowledge scores observed among laboratory students may therefore reflect differences in expected clinical exposure and curricular emphasis compared with clinically oriented disciplines. Limited clinical exposure to epilepsy surgery during undergraduate training may contribute to insufficient awareness and delayed recognition of appropriate surgical referral candidates. The uncertainty regarding epilepsy surgery risk observed among participants may reflect limited exposure to contemporary epilepsy surgery outcomes during undergraduate education.

Several previous studies have evaluated the knowledge and attitudes about epilepsy surgery among neurologists and other healthcare professionals [11, 15–17] Although many studies have assessed the knowledge and attitudes of medical students toward epilepsy, only a few have specifically addressed awareness of epilepsy surgery [12, 14, 18]. Surgery is an effective, evidence‐based method for treating epilepsy, but there is often a delay in referral to epilepsy surgery centers. This delay results in many years lost, during which patients suffer from various medical and social adverse events and face potential mortality. Therefore, we believe that introducing the concept of epilepsy surgery as early as possible in the medical curriculum and allied healthcare education is essential. This early introduction can improve knowledge and change attitudes toward surgical treatment of epilepsy, ultimately leading to earlier referral [12–14]. Early referral for epilepsy surgery evaluation is particularly important because prolonged uncontrolled seizures are associated with cognitive decline, psychosocial impairment, reduced quality of life, and increased mortality risk, including SUDEP. Current ILAE recommendations emphasize that all patients with DRE should undergo evaluation at a specialized epilepsy center to determine surgical candidacy. Therefore, inadequate awareness among future healthcare providers may contribute to delayed referral practices and underutilization of potentially life‐changing surgical treatment options. Our findings are consistent with recent international evidence demonstrating persistent knowledge gaps regarding epilepsy surgery and referral practices among healthcare professionals, highlighting the continued need for targeted educational initiatives to improve awareness and facilitate timely referral of patients with DRE [19–21].

A study conducted among medical students in Slovenia showed that the term “DRE” was known to 72.3% of students, compared with only 29.9% in our sample. However, when stratified by specialty, 56% of medical students in our study knew the term, whereas only 25.8% of nursing students, 15.1% of pharmacy students, and 6.2% of laboratory students were aware of it [12].

Approximately one‐quarter of participants (25.8%) were aware that epilepsy could be treated surgically. Awareness was highest among medical students, of whom 50.7% had heard about epilepsy surgery, compared with 23.8% of nursing students, 10.9% of pharmacy students, and 5% of laboratory students. This finding is comparable with that reported among medical students at Umm Al‐Qura University in Saudi Arabia (49.6%) but lower than the level reported in Slovenia (74.5%) [12, 14]. In our sample, the year of study did not significantly affect the knowledge level; however, in Slovenia, the general level of knowledge improved in the later years of study [12]. In the Umm Al‐Qura University study, 24.4% of students were familiar with the term “DRE,” and more than half of the students indicated that they would refer patients with DRE to a neurosurgeon and neurologist [15].

This finding is similar to our medical students, where 64% demonstrated this level of awareness, compared with 29.1% of nursing students, 10.9% of pharmacy students, and 12.5% of laboratory students who answered correctly.

Consistent with our study, more than half of the medical students did not know what type of epilepsy could be surgically treated (66.1% in the Umm Al‐Qura study compared with 60% of our medical student sample) [14].

Knowledge regarding surgically treatable epilepsy syndromes remained limited across all disciplines, particularly among nonmedical trainees. Mesial temporal sclerosis is one of the most common pathologies encountered among patients with epilepsy, and knowledge about it is important for general practitioners. In our study, 89.4% of participants had never heard of the term “mesial temporal” or “hippocampal sclerosis” (81.3% of medical students, 87.4% of nursing students, 96.6% of pharmacy students, and 97.5% of laboratory students). Similarly, in the Slovenia study, the level of knowledge about this condition was poor among medical students [12].

In a Saudi study among neurologists, only 57.5% of the neurologists had a sufficient level of knowledge regarding epilepsy surgery, and more than half (52.8%) had a positive attitude toward epilepsy surgery. There was a positive correlation between knowledge level and attitude [15].

A study conducted among Swedish neurologists revealed a generally positive attitude toward epilepsy surgery, even though most neurologists tried three or more ASM before considering surgical intervention. Only 66% of the neurologists correctly defined the term “DRE” [16]. A Canada‐wide survey found that nearly half of the neurologists did not correctly define DRE [17].

A study conducted at our institution in 2018 regarding knowledge and attitudes toward epilepsy showed that 95.3% of respondents had heard or read about epilepsy, 34.2% knew someone with epilepsy, and 53.5% had witnessed someone having a seizure. Furthermore, 46.4% of the medical students believed that surgical management is an option for refractory seizures, compared with 50.7% of medical students in the current sample [18]. Comparison with our institution′s previous 2018 study suggests only minimal improvement in awareness regarding epilepsy surgery over time, with awareness increasing from 46.4% to 50.7% among medical students. This limited change over several years further supports the need for more structured and standardized educational interventions addressing epilepsy surgery and DRE referral practices.

Additionally, it was observed that the knowledge level improved during the clinical years compared with the preclinical years in the previous study. However, in the current study, there was no significant difference in the overall knowledge score between preclinical and clinical years. This may suggest that in our curriculum, epilepsy knowledge is introduced progressively throughout the academic years, whereas a similar progression in surgical knowledge is lacking.

There were significant associations between the level of knowledge and both the participant′s specialty (*p* < 0.0001) and whether the participant had completed a neurology or neurosurgery rotation (*p* < 0.0001). Notably, only three participants achieved high knowledge scores despite the presence of neurology or neurosurgery rotation exposure among some students. This finding may suggest that epilepsy surgery referral criteria and surgical management concepts are not consistently or systematically emphasized within current undergraduate clinical training materials.

When examining participants with high knowledge scores, the only three participants who achieved high scores were medical students. Among participants with low scores, only 22.5% were medical students, wheras 77.5% were nonmedical students. In contrast, among participants with moderate scores, 61.8% were medical students, and 38.2% were nonmedical students. The total knowledge score was highest among medical students and lowest among laboratory students (12.33 ± 4.04 vs. 9.85 ± 1.52). This study is among the few to evaluate awareness of epilepsy surgery across multiple healthcare disciplines rather than focusing exclusively on medical students or physicians. Multidisciplinary epilepsy care increasingly involves nurses, pharmacists, and allied healthcare professionals who may contribute to patient counseling, medication monitoring, perioperative coordination, long‐term follow‐up, and reinforcement of referral recommendations. Improving baseline awareness across healthcare disciplines may therefore support earlier recognition of DRE and strengthen collaborative epilepsy care pathways. A recent nationwide study from Mexico similarly demonstrated persistent deficiencies in knowledge regarding DRE definitions and epilepsy surgery referral practices among physicians, with lower awareness observed among older practitioners. These findings further emphasize the importance of improving education regarding epilepsy surgery and DRE referral criteria during undergraduate medical training and early professional development [22]. The findings of this study may also be relevant to other low‐ and middle‐income countries (LMICs) where epilepsy surgery remains underutilized and educational exposure to epilepsy referral pathways may be limited. Similar barriers related to awareness, delayed referral, and insufficient multidisciplinary training have been reported internationally. A recent quasiexperimental study showed that structured peer education significantly improved epilepsy knowledge scores, attitudes toward epilepsy, and confidence in seizure first aid management among students [23]. Similarly, web‐based educational interventions for future healthcare professionals have been shown to improve epilepsy‐related knowledge and preparedness [24], while targeted educational programs for nursing students have also demonstrated significant improvements in epilepsy knowledge, attitudes, and first aid approaches [25]. These findings further support the importance of incorporating structured epilepsy education initiatives into undergraduate healthcare training programs to bridge the gap between theoretical knowledge and clinical preparedness.

Our study has several limitations. Since the data were gathered through a self‐reported survey, there is a possibility of recall bias; participants may have consulted external resources while completing the survey, potentially introducing response bias. Moreover, because the questionnaire was distributed broadly through electronic platforms and student communication groups, the exact number of students who received the survey invitation could not be determined, and the response rate could not be calculated. Additionally, we only included medical students and interns from King Abdulaziz University, which may limit the generalizability of our findings to other populations. Although we did not conduct a pilot test of the survey, the survey was developed based on previous studies conducted among neurologists and medical students. In addition, the questionnaire was not specifically reviewed by epileptologists or neurologists specializing in epilepsy surgery, which may have influenced the precision of certain questionnaire items. Furthermore, the study evaluated students and interns rather than practicing healthcare professionals; therefore, the findings may not fully generalize to practicing physicians, nurses, pharmacists, or laboratory professionals. In addition, formal construct validation, pilot testing, and reliability analysis using Cronbach′s alpha were not performed for the questionnaire, which may limit the psychometric robustness of the study instrument.

### 4.1. Recommendations

Based on the findings of this study, integrating structured educational content on epilepsy surgery into undergraduate medical and allied healthcare curricula may improve awareness of surgical treatment options and facilitate earlier referral of patients with DRE. Clinical exposure through neurology and neurosurgery rotations, case‐based discussions, interdisciplinary teaching sessions, and epilepsy‐focused workshops may further enhance students’ understanding of epilepsy surgery and its indications. Future multicenter studies involving multiple universities across Saudi Arabia are recommended to evaluate national awareness levels and assess the effectiveness of educational interventions.

## 5. Conclusion

Most healthcare students and interns demonstrated limited knowledge and awareness regarding epilepsy surgery, particularly concerning surgical indications, DRE, referral timing, and available treatment modalities. Higher knowledge scores were observed among medical students and participants who had completed neurology or neurosurgery rotations, likely reflecting differences in clinical exposure and curricular emphasis across healthcare disciplines.

Strengthening baseline knowledge and awareness of epilepsy surgery across multiple healthcare disciplines may improve multidisciplinary epilepsy care pathways, facilitate earlier recognition and referral of patients with DRE, and ultimately contribute to improved patient outcomes and collaborative clinical practice.

Integrating structured educational content, interdisciplinary teaching sessions, and greater clinical exposure to epilepsy surgery within healthcare training programs may help address current educational gaps and improve future epilepsy care delivery.

## Funding

No funding was received for this manuscript.

## Conflicts of Interest

The authors declare no conflicts of interest.

## Supporting information


**Supporting Information** Additional supporting information can be found online in the Supporting Information section. STROBE statement—Checklist of items that should be included in reports of cross‐sectional studies.

## Data Availability

The data that support the findings of this study are available from the corresponding author upon reasonable request.
